# Possibilities of using the European bison (*Bison bonasus) *epididymal spermatozoa collected post-mortem for cryopreservation and artificial insemination: a pilot study

**DOI:** 10.1186/1477-7827-9-31

**Published:** 2011-03-08

**Authors:** Roland Kozdrowski, Wojciech Niżański, Andrzej Dubiel, Wanda Olech

**Affiliations:** 1 Department of Reproduction and Clinic of Farm Animals, Wrocław University of Life and Environmental Science, Wrocław, Poland; 2 Department of Genetics and Animal Breeding, Warsaw University of Life Sciences, Warsaw, Poland

## Abstract

**Background:**

European bison is the largest mammal in Europe with the population of approximately 4000 individuals. However, there is no report of post-mortem spermatozoa collection and cryopreservation from this species and the aim of this study was to test if the epididymal spermatozoa collected post-mortem from European bison are suitable for cryopreservation and artificial insemination (AI).

**Methods:**

Epididymides were collected post-mortem from two European bison bulls at age of 8 (bull 1) and 11 year (bull 2). Epididymal sperm was harvested by making multiple incisions in caudae epididymidis, which were then rinsed with extender. The left epididymis of bull 1 was rinsed with BioXcell (IMV, France), whereas the right epididymis of bull 1 and the right and left epididymides of bull 2 were rinsed with the extender based on Tris, citric acid, glucose, egg yolk, glycerol, antibiotics and distilled water (extender II). The diluted semen was cooled to 5 degrees C, and frozen in liquid nitrogen vapour. Then, properties of the frozen/thawed semen were examined with the use of computer-assisted semen analysis system, and thirty cows and nine heifers of domestic cattle were artificially inseminated.

**Results:**

Motility of fresh spermatozoa collected from the right epididymis of bull 1 was 70% (spermatozoa diluted with extender II), and from the left one was 60% (spermatozoa diluted with BioXcell), whereas motility of fresh spermatozoa collected from bull 2 was 90% (spermatozoa diluted with extender II). Spermatozoa motility just after thawing were 11 and 13% in bull 1, respectively for spermatozoa collected from the left and right epididymis and 48% in bull 2. As a result of AI of domestic cows and heifers with the frozen/thawed European bison spermatozoa, two pregnancies were obtained in heifers. One pregnancy finished with a premature labour after 253 days of pregnancy, and the second one after 264 days of pregnancy.

**Conclusions:**

This is the first report showing pregnancy in the domestic cattle following AI with frozen-thawed European bison spermatozoa collected post-mortem. The protocol of spermatozoa collection, dilution, and cryopreservation presented in this paper may be useful for the creating genetic resource bank in the European bison.

## Background

The best way for conservation of endangered animal species is protection of their natural environment. However, the natural environment, as a result of human activities, is considerably shrinking which restricts free migration of individuals and hampers exchange of the genetic material between isolated populations. This situation refers also to the European bison, the largest mammal in Europe, which population, now, amounts to approximately 4000 individuals living in natural environment and in captivity. It ought to be stressed that the European bison population is highly inbred as all currently living individuals come from 12 ancestors [1]. One of the ways in which biodiversity may be protected is to use assisted reproductive techniques in order to collect the genetic material from endangered mammalian species (creating the genetic resource banking) and consequently introduce the assisted reproduction techniques into the *in situ *and *ex situ *conservation programs [2-5]. Semen cryopreservation and artificial insemination (AI) are one of the above-mentioned methods. Semen cryopreservation enables creation of a semen bank, and by means of AI, it seems possible the infusion of new genetic material to the isolated populations. Nevertheless, the introduction of AI requires research on semen collection techniques from endangered species (from live or dead animals) and also requires research on semen cryopreservation, and the first successful trails of semen collection with the use of electroejaculation method from live male of European bison and semen cryopreservation have only been described recently [6]. The introduction of AI into European bison also requires research on a female reproductive physiology, working out of the protocols for synchronization of oestrus cycle and ovulation, as well as a suitable site of semen deposition into genital female tract, and a number of spermatozoa and a volume of insemination dose should also be specified (these issues have not been worked out for the European bison yet). As a first step, the aim of this study was to show the proof of principle of post-mortem collection and cryopreservation of epididymal sperm of European bison, with post-thaw fertilizing capacity.

## Methods

### Collection and processing of spermatozoa

At the end of November, the testes were collected post-mortem from the two European bison bulls at the age of 8 (male bison 1) and 11 year (male bison 2), shot according to the Polish law. Within a few minutes from the death of the animal testes were collected and transported at a temperature of approximately 15°C to the laboratory within approximately 1 hour, where next, epididymides were dissected from the testes. Epididymal sperm was harvested by making multiple incisions in caudae epididymidis, which were then rinsed with 6 ml of extender warmed up to 33°C. The left epididymis of bull 1 was rinsed with BioXcell^® ^(IMV, France), a commercial extender available for bull semen (this extender contains soybean extract, a substitute for egg yolk and glycerol), whereas the right epididymis of bull 1, and the right and left epididymides of bull 2 were rinsed with the extender of the following composition: Tris (2.4 g), citric acid (1.4 g), glucose (0.8 g), egg yolk (20%, v/v), penicillin (5000 iu), streptomycin (100 mg), glycerol (4%, v/v) and distilled water to 100 ml (extender II). After 10 min of incubation at a temperature of 33°C, a percentage of motile spermatozoa was assessed under a light microscope equipped with a 37°C thermostable table at 200×. The spermatozoa concentration per unit volume was calculated by the cytometric method in a Thom-Zeiss chamber.

Next, the subsequent dilution was carried out (at a temperature of 33°C) with the use of the same extenders. The final volume of the added extender depended on the spermatozoa motility and number of collected spermatozoa. In the case of the right epididymis of male bison 1, no subsequent dilution was performed, as spermatozoa concentration after initial rinsing was low (32 × 10^6^/ml), which, finally, allowed to place only 8 × 10^6 ^spermatozoa into a 0.25 ml straw. The spermatozoa from the left epididymis of male bison 1 were finally diluted, up to an amount of 25 × 10^6 ^of motile spermatozoa in a volume of 0.25 ml. The spermatozoa from the right and left epididymides of male bison 2 were pooled (due to the use of the same extender for spermatozoa collected from left and right epididymides and the same spermatozoa motility), and finally diluted up to 35 × 10^6 ^of motile spermatozoa in a volume of 0.25 ml.

### Sperm cryopreservation and thawing

Next, the diluted semen was placed in small containers, which were dipped into water bath of 33°C and placed in a fridge at 5°C for 4 h. After cooling to 5°C, the diluted semen was packed into 0.25 ml Cassou straws, and the free end of each straw was sealed with polyvinyl alcohol. Next, the straws were placed horizontally 5 cm above the surface of liquid nitrogen for 15 min, and then plunging into the liquid nitrogen. A few weeks after placing the straws in the liquid nitrogen, the straws were thawed in a water bath of 37°C for 30 s.

### Post-thaw sperm evaluation using computer-assisted semen analysis (CASA)

For CASA analysis, 1 ml of PBS was slowly added to 0.25 ml thawed semen in 2 ml tubes at 37°C. The tubes were then incubated at 37°C for 10 and 90 min, and the spermatozoa motility and velocity were subsequently evaluated with HTM IVOS version 12.2 (Hamilton-Thorne Biosciences Beverly, MA, USA). For the assessment, 4 μl of diluted semen was placed in a Leja 4 analysis chamber of a depth of 19.7 μm (Leja Products B.V., Holland). Slides were mounted on a stage warmer at 37°C.

The following motility and velocity characteristics were evaluated: average path velocity (VAP, μm/sec), straight velocity (VSL, μm/sec), curvilinear velocity (VCL, μm/sec), amplitude of lateral head displacement (ALH, μm), beat cross frequency (BCH, Hz), motility (MOT, %) and progressive motility (PMOT, %). The definitions for each of these characteristics are presented in another paper [7]. The CASA values for European bison sperm analysis were as a starting parameters for bull spermatozoa: frame rate (60 Hz), frames acquired (30), minimum contrast (80), minimum cell size (5 pixels), low VAP cut-off (30 μm/sec) and low VSL cut-off (15 μm/sec).

### Artificial insemination and pregnancy diagnosis

For AI only spermatozoa collected and frozen/thawed from bull 2 were used, which were characterized by a high percentage of motile spermatozoa after thawing (see the Results). A total of 30 domestic Holstein Frisian cows being in spontaneous oestrus or synchronized with an Ovsynch protocol were inseminated. Cows subjected to Ovsynch protocol were treated with 0.02 mg of buserelin (GnRH analogue, Receptal^®^, Intervet GmbH, Germany, i.m.) on Day 0. Next on Day 7, cows were treated with 25 mg of dinoprost (PGF_2_α, Dinolytic^®^, Pharmacia, Belgium, i.m.), and on Day 9, cows received a second dose of buserelin. Unfortunately, no cows have been pregnant after AI with frozen/thawed European bison sperm (see the Results), therefore a decision of insemination of heifers was made, because generally the heifers are characterized by better fertility rate compared to the cows [8]. A total of 9 heifers were synchronized with an Ovsynch protocol as described above. In all cows and heifers, insemination was performed 12-18 h from the beginning of the heat (only females which exhibit oestrus signs after synchronization with Ovsynch protocol were inseminated). The semen for AI was thawed in the same way as described above and was slowly deposited with Cassou gun into uterine body (cows), or into the uterine horn ipsilateral to the ovary on which follicle was detected by rectal palpation (heifers). Each female received one straw of frozen/thawed spermatozoa per insemination. Additionally heifers were inseminated again 12 h after the first insemination. Pregnancy diagnosis was carried out by palpation of the uterus 6 weeks after AI.

## Results

After 10 min incubation, motility of the fresh spermatozoa collected from the right epididymis of bull 1 was 70% (spermatozoa diluted with extender II), and from the left one was 60% (spermatozoa diluted with BioXcell^®^), whereas motility of the fresh spermatozoa collected from bull 2 (from right and left epididymides) was 90% (spermatozoa diluted with extender II). The results regarding motility and velocity characteristics of frozen/thawed spermatozoa observed after 10 and 90 min incubation at a temperature of 37°C are presented in Table 1 and 2, respectively. The information presented in Table 1 and 2 shows that motility of spermatozoa collected from bull 2 was a few times higher compared to bull 1. After 90 min incubation, a percentage of motile spermatozoa decreased to 8% in bull 2 and in bull 1 to 2% (spermatozoa diluted with extender II) and 1% (spermatozoa diluted with BioXcell^®^).

**Table 1:** Properties of frozen/thawed semen evaluated with CASA immediately after thawing

Trial	MOT (%)	PMOT (%)	VAP (mm/s)	VSL (mm/s)	VCL (mm/s)	ALH (mm)	BCF (Hz)	LIN (%)	STR (%)
Bull 1 (spermatozoa diluted with BioXcell^®^)	11.00	5.00	94.20	66.10	179.10	9.10	20.20	38.00	67.00
Bull 1 (spermatozoa diluted with extender no II)	13.00	8.00	88.90	64.40	171.80	7.50	17.30	41.00	72.00
Bull 2 (spermatozoa diluted with extender no II)	48.00	20.00	140.00	86.40	270.40	10.50	23.60	34.00	61.00

**Table 2:** Properties of frozen/thawed semen evaluated with CASA 90 min after incubation at 37°C

Trial	MOT (%)	PMOT (%)	VAP (mm/s)	VSL (mm/s)	VCL (mm/s)	ALH (mm)	BCF (Hz)	LIN (%)	STR (%)
Bull 1 (spermatozoa diluted with BioXcell^®^)	1	0.00	30.07	27.02	88.90	1.0	17.00	37.00	70.00
Bull 1 (spermatozoa diluted with extender no II)	2	0.00	41.60	28.20	90.20	1.0	16.00	34.00	69.00
Bull 2 (spermatozoa diluted with extender no II)	8	2	62.80	41.30	143.60	6.1	30.60	32.00	66.00

**Figure 1:**
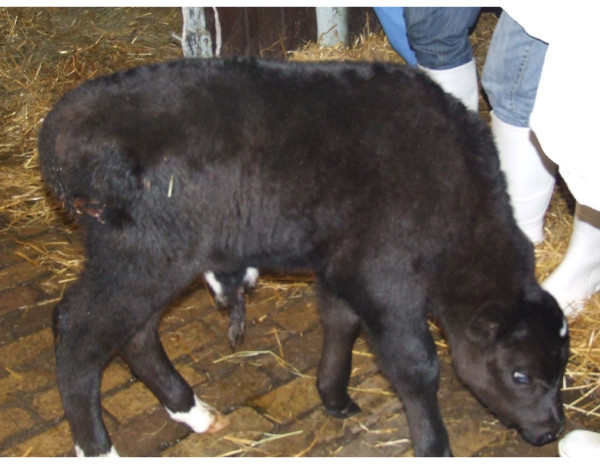
**Two days old male hybrid**.

**Figure 2:**
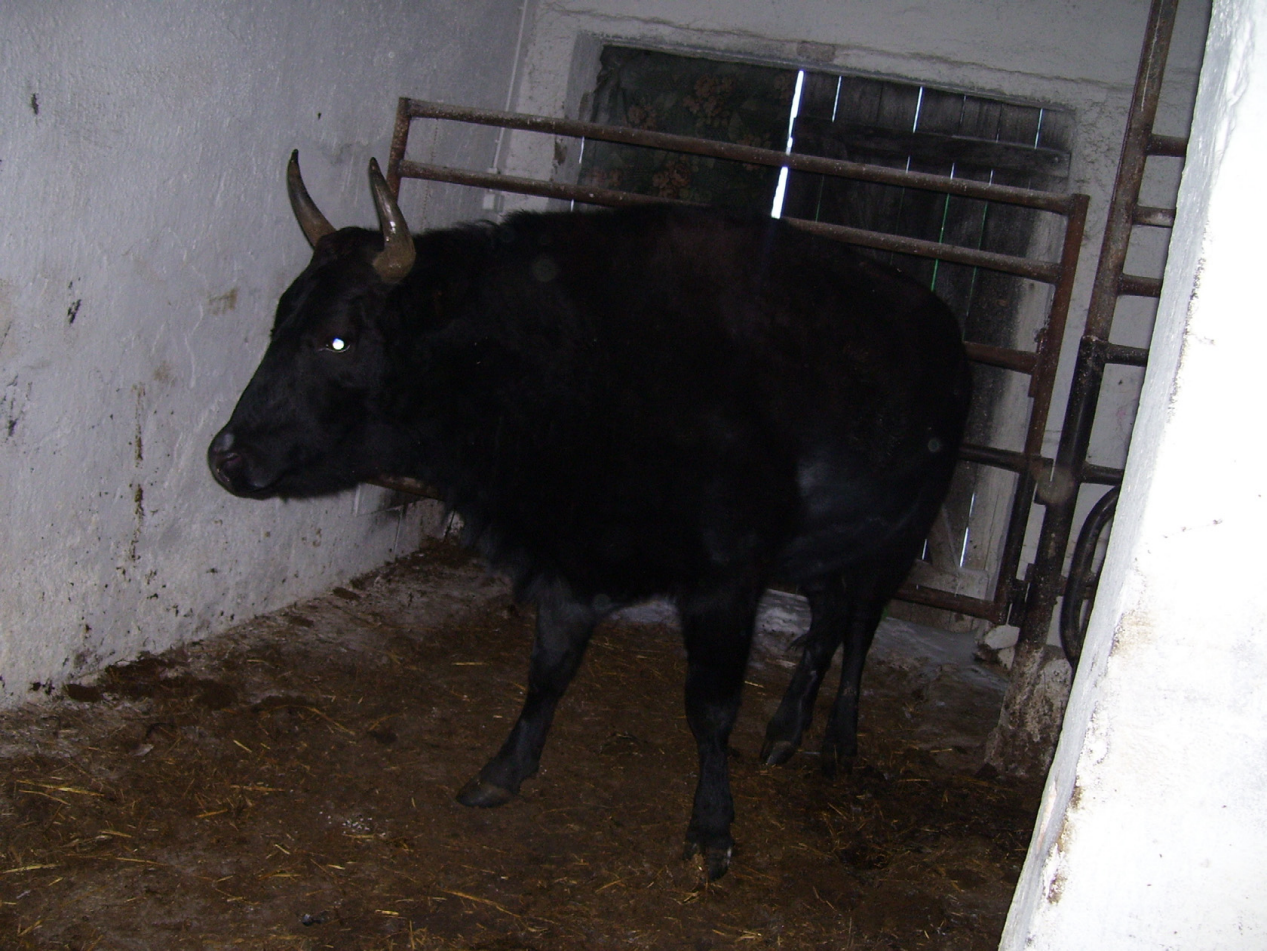
**Two year old female hybrid**.

Six weeks after AI, pregnancy was detected in none of the cows, yet, two of the nine heifers were pregnant. In one of the heifers, labour started on 253 day after AI (AI day 0). However, due to a oversize of the foetus, the labour was delivered through Caesarean operation. A male calf (Figure 1) died 7 days after the birth due to pneumonia. The other heifer gave birth to a female calf 264 days after AI (AI day 0) (Figure 2).

## Discussion

A few studies on hybridisation of European bison and domestic cattle were carried out in Poland in the second half of the last century [9,10]. In above mentioned studies, European bison females were mated with a male of the domestic cattle, or females of the domestic cattle were mated with a male of European bison without the use of AI. It was shown, that conception rate in the crosses of European bison male and domestic cows was relatively low, and amounted to 14%, and it was also accompanied by a high percentage of pregnancy losses reaching 30%, especially, for a combination of European bison male and domestic cow [9]. According to the authors' knowledge the first effective AI of the domestic cattle with the European bison spermatozoa collected post-mortem and cryopreserved have been described in this paper. The aim of this study was to test if the epididymal spermatozoa collected post-mortem from European bison are suitable for cryopreservation and AI. Due to the fact that it was impossible to perform AI on the female European bison, the insemination was carried out on the females of domestic cattle. As mentioned above, mating the domestic cows with the European bison is little effective, therefore the fact of obtaining offspring through AI of the domestic cattle with the European bison spermatozoa collected post-mortem from epididymides, and frozen/thawed should be considered as an effective way of European bison spermatozoa collection and preservation, which is the basis for creation of a semen bank for the biggest European mammal.

It was shown that, epididymal spermatozoa collected post-mortem from domestic bulls are suitable for cryopreservation and AI [11], and that epididymal spermatozoa can be used in *in vitro *production of embryos [12]. In some wildlife species it was also shown, that epididymal spermatozoa collected post-mortem can be successfully cryopreserved and used in AI programme [13-15]. Santiago-Moreno and colleagues [14] described a successful heterologus AI of domestic goat with cryopreserved epididymal spermatozoa collected from Spanish ibex, and they concluded, that heterologus *in vivo *fertilization is a useful method to evaluate the fertilizing capacity of sperm in rare or wild species. A low conception rate observed in this study can be explained, first of all, by interspecies insemination. In addition, it should be highlighted that the inseminated cows were characterized by an average milk yield at a level of 9000 kg per lactation, and a relatively low conception rate at a level of 30-40% is usually observed in herds with such high milk production [16]. On the other hand, heifers are characterized by better fertility than cows [8]. Unfortunately, it was not possible to perform AI in a different, more fertile breed of cattle however, conception rate in the crosses of European bison male with polish red and black-white lowland cows was also low and amounted to 14% [9].

Additionally, in this experiment semen was deposited in heifers into the uterine horn, ipsilateral to the ovary on which follicle was detected, and two inseminations within 12 h was applied. Perhaps the insemination into the uterine horn and double insemination within 12 h significantly influenced the experiment results, because laboratory examination of the frozen/thawed European bison spermatozoa collected post-mortem showed, that percentage of motile spermatozoa significantly decreased after 90 min incubation at a temperature of 37°C, which may suggest a short survival rate of cryopreserved spermatozoa in a reproductive tract of the female. An examination for pregnancy was carried out six weeks after AI, therefore it cannot be excluded that part of the females got pregnant but an early embryonic death could took place.

The laboratory examination of the European bison epididymal spermatozoa collected post-mortem from two bulls showed significant individual differences in spermatozoa motility, both before freezing and after thawing. Nevertheless, it was shown that it was possible to collect post-mortem spermatozoa from the European bison characterized by a high motility score. It should be stressed, that the material for the examination was collected at the end of November, during non-breeding season of the European bison, which occurs from August to October [17]. There is not any information in the literature on an influence of a season on the quality of the European bison semen, nevertheless, a significant influence of the season was showed for other wild mammals living in the same latitude [18,19]. We could not collect epididymal spermatozoa during breeding season, because European bison were permitted to cull in November. In spite of the promising results obtained in this study, disadvantages of post-mortem spermatozoa collection from epididymises should also be pointed out. One of the negative aspects of such procedure is the fact that spermatozoa may be collected only once from a given male, and the epididymal spermatozoa may be collected only from culled European bison, which significantly limits the number and quality of collected spermatozoa and, as a consequence, the number of insemination doses produced. Additionally, there is a risk of an irretrievable loss of spermatozoa when a mistake is made during preparation of the samples.

In our study, we used two extenders for semen dilution. One of them, BioXcell^®^, does not contain any animal proteins, which is designed to eliminate alleged health risks of insemination media containing milk or egg yolk. The BioXcell^® ^extender was applied for dilution of spermatozoa collected from the left epididymis of male bison 1. Spermatozoa motility after dilution in this extender was 10% lower compared to spermatozoa collected from the same male and diluted in extender II. Similarly, after thawing, motility of spermatozoa isolated from other epididymis and diluted with the BioXcell^® ^extender was lower compared to motility of spermatozoa collected from the same male, and diluted with extender II (Table 2). It ought to be stressed that the above-mentioned observation regards only one bull, and spermatozoa collected from him were characterized generally by a low percentage of spermatozoa motility. However, Herold and colleagues [20] showed that epididymal spermatozoa collected from African buffalo (*Syncerus caffer*) and diluted with Triladyl^® ^are characterized by better motility than spermatozoa diluted with AndroMed^® ^(totally defined semen extender free of animal products) after freezing/thawing. On the other hand, Pérez-Garnelo and colleagues [6], examining semen collected with the use of the electroejaculation method from one European bison did not show an influence of the extender (Triladyl^® ^and synthetic extender based on Triladyl^® ^in which egg yolk was replaced by soybean lipids) on post-thaw individual motility, quality of movement and sperm morphology. However, they observed a higher number of spermatozoa with intact acrosomes, intact membranes and viable sperm frozen in Triladyl^® ^compared to the synthetic extender. We suspect, that individual characteristics of a bull from which spermatozoa were collected had a greater influence on the spermatozoa motility than a type of extender used, and the studies which focus on determination of usefulness of particular extenders for cryopreservation of epididymal spermatozoa collected post-mortem from the European bison require examination based on a bigger amount of material.

## Conclusions

Summing up, it should be stated that it is possible to collect epididymal spermatozoa from the European bison suitable for cryopreservation and AI, and the protocol of spermatozoa collection, dilution, and cryopreservation presented in this paper may be useful for the creating genetic resource bank in the European bison. On the basis of the presented results, it may be recommended that one insemination dose of European bison male should contain 35 × 10^6 ^of motile spermatozoa (before freezing) in a volume of 0.25 ml. Nevertheless, the use of frozen/thawed semen for insemination of the European bison females requires a research on female reproductive physiology, working out of the protocols for synchronization of oestrus cycle and ovulation, as well as a suitable site of semen deposition into genital female tract should also be specified.

## List of abbreviations

AI: artificial insemination; CASA: computer-assisted semen analysis.

## Competing interests

The authors declare that they have no competing interests.

## Authors' contributions

RK, WN and AD participated in spermatozoa collection, freezing and evaluation. RK was also responsible for artificial insemination, caesarean operation and the writing the manuscript. WO arranged European bison males for this study. All authors read and approved the final manuscript.
